# Obesity prevention: the role of policies, laws and regulations

**DOI:** 10.1186/1743-8462-5-12

**Published:** 2008-06-05

**Authors:** Boyd A Swinburn

**Affiliations:** 1 WHO Collaborating Centre for Obesity Prevention, Deakin University, 221 Burwood Highway, Melbourne, 3125, Australia

## Abstract

The commercial drivers of the obesity epidemic are so influential that obesity can be considered a robust sign of commercial success – consumers are buying more food, more cars and more energy-saving machines. It is unlikely that these powerful economic forces will change sufficiently in response to consumer desires to eat less and move more or corporate desires to be more socially responsible. When the free market creates substantial population detriments and health inequalities, government policies are needed to change the ground rules in favour of population benefits.

Concerted action is needed from governments in four broad areas: provide leadership to set the agenda and show the way; advocate for a multi-sector response and establish the mechanisms for all sectors to engage and enhance action; develop and implement policies (including laws and regulations) to create healthier food and activity environments, and; secure increased and continued funding to reduce obesogenic environments and promote healthy eating and physical activity.

Policies, laws and regulations are often needed to drive the environmental and social changes that, eventually, will have a sustainable impact on reducing obesity. An 'obesity impact assessment' on legislation such as public liability, urban planning, transport, food safety, agriculture, and trade may identify 'rules' which contribute to obesogenic environments. In other areas, such as marketing to children, school food, and taxes/levies, there may be opportunities for regulations to actively support obesity prevention. Legislation in other areas such as to reduce climate change may also contribute to obesity prevention ('stealth interventions'). A political willingness to use policy instruments to drive change will probably be an early hallmark of successful obesity prevention.

## The obesity epidemic

Historically, obesity prevalence rates have been low and relatively unchanging until about 20–30 years ago. In countries where regular monitoring of population heights and weights have been in place for several decades, a fairly consistent upward inflection was seen in the prevalence of obesity from about the early 1980s in children [1] and adults [2]. Surveys from around the world now confirm that obesity has reached pandemic proportions, with many developing countries now struggling under the double burden of continuing high rates of infectious diseases and rising rates of cardiovascular diseases, diabetes, and obesity [3]. No country has managed to reduce the burden of obesity using public health approaches. The possible exception is Singapore with its Trim and Fit program for children [4], although aspects of the program have recently been revised because of the risk of stigmatising obese children by singling them out for extra exercise sessions.

## Drivers of the increasing obesity epidemic

The drivers of this pandemic that is now affecting rich and poor countries alike must be global in nature and relatively recent in onset. While biological hard-wiring explains the potential for the development of obesity, it cannot explain the secular trends in obesity prevalence. Humans have, for good survival reasons, evolved a biology that is designed to maximise energy intake and minimise physical activity. We seek and enjoy good tasting food (especially sweet, fatty and salty foods) and we seek to reduce the effort needed to do work (by designing machines and technology to do it for us). While these are powerful factors, our biology has not changed over the last 30 years. What has changed dramatically is the environment around us – especially the easy availability of foods and energy-saving machines that feed those biological desires. It is the increasingly obesogenic environments which are promoting especially excessive energy intake but also reduced physical exertion that are driving secular trends [5].

Environments that affect our behaviours can be broadly categorised into physical (what is or is not available), economic (the financial factors), policy (the 'rules'), and socio-cultural (the attitudes, beliefs, perceptions, values and norms of the societal or cultural group) [6]. This has been a helpful and robust framework for scanning obesogenic environments and creating comprehensive lists of potential elements external to individuals that may influence behaviours. However, this list does not tell us which of these types of environmental factors are likely to be dominant as the drivers of the epidemic or as the potential solutions that are urgently needed to turn the epidemic around. The proposal in this paper is that the dominant environmental drivers of obesity are economic and that the dominant solutions will need to be policy-based. Before examining these two components in more detail, the other aspects of obesogenic environments are placed in the context as likely contributors to the epidemic.

The built urban environment has many physical features which influence physical activity levels: transport systems, recreation facilities and spaces, aesthetics, street design, land use, access to destinations like shops and schools and so on [7]. In many cities, these features are obesogenic although, being structural, they are usually quite slow to change and are therefore likely to be underlying factors rather than triggering factors for the recent rise in obesity prevalence. A much more rapid environmental change that promotes physical inactivity has been the flood of technology that provides increasing numbers of labour-saving devices and passive entertainment options. However, arguably the biggest obesogenic environmental change has been the increased availability and promotion of cheap, energy-dense foods [8].

There are some aspects of the policy environment (the 'rules') that may be inadvertently contributing to obesogenic environments and these include the increasing reach of public liability laws (for example, causing schools to lock their grounds after hours), farm policies in the US and Europe that subsidise fat and sugar production and keep fruit and vegetable prices high, and urban planning regulations which promote single rather than mixed land use in cities.

The socio-cultural environments that influence food, eating patterns and physical activity vary enormously across populations and these influences undoubtedly explain many of the differences in obesity prevalence among populations and sub-populations [9,10]. For example, cultures may differ in the expectations that they place on hosts (to over-provide food) and guests (to over-consume food), the appropriateness for girls and women to be physically active, the status of certain foods or dishes, or the beliefs in the value of food and physical activity for health. This may mean that the socio-cultural differences between groups may confer a relative predisposition to or protection from weight gain when the group is exposed to a modern obesogenic environment. The variation of obesity prevalence from less than 1% (India) to nearly 60% (Tonga) [2] suggests socio-cultural differences are very important. However, these are probably best thought of as moderating factors either enhancing or cushioning the effects of the real drivers of the obesity epidemic. The concept of 'socio-cultural predisposition' to obesity is more akin to 'genetic predisposition' implying an underlying state which needs change in context to become manifest. George Bray famously stated that 'genes load the gun, but the environment pulls the trigger' [11]. This could now be updated to 'genes and culture load the gun, but the economic environment pulls the trigger'.

## Obesity as a commercial success but market failure

As mentioned, the two broad obesogenic changes in the environment that have noticeably increased in the past 30 years have been the upsurge in obesogenic food and machines. Energy-dense foods and beverages are now readily available, highly promoted, and low-cost [12,13]. There are two types of machines that reduce energy expenditure: labour-saving devices such as cars, computers, and occupational and domestic machines, and; passive entertainment machines such as television, video, and electronic games [14,15].

The list of commercial products which promote excessive energy intake or decreased energy expenditure is very long and those products are usually heavily marketed (cars and foods are the two highest advertised products [16]). By comparison, the list of products that would maintain a healthy energy intake (eg fruit and vegetables) or increased physical activity (eg bicycles) is much shorter and their marketing budgets are tiny [17].

The driving forces behind the consumption, indeed over-consumption, of these obesogenic products are commercial (profit incentives), and market economies are now the backbone of all successful economic systems. A high consumption constitutes a 'commercial success' because the sellers make a profit, but to be considered a 'market success' both sellers and buyers need to gain from the transaction [18]. The buyers, in the short term, certainly do gain. They get good tasting food at low prices and lots of it if they eat at buffet restaurants or buy two-for-one or up-size their serving to get better value for money. At a relatively low cost, they get enjoyable entertainment, new energy-saving domestic appliances to open tin cans or blow the leaves from their driveways, and more automatic features in their car. At one level, this is a 'market success' because customers are apparently making free choices to satisfying their needs and desires – or in economic jargon making 'preference decisions to maximise their utility'. However, in the long term, people do not like to be obese – it is not their 'preference' nor does it give them high utility (in this case, health and quality of life). Humans are notoriously prone to choosing more for instant gratification than for long term benefits and they are also prone to the marketing pressures which 'create' the desires in the first place [19]. All these points particularly apply to children because they are much more dominated by short term desires than long term outcomes. Commercial drivers may also explain, in part, the increasing inequalities seen with obesity. People living in lower income areas often have less access to public transport and recreation facilities and are more heavily targeted by fast food restaurants [20].

The increasing obesity prevalence and inequalities can, therefore, be described as a 'market failure' because the free market system is failing to promote and sustain long-term individual and social goals [17]. In orthodox economic theory, 'market failure' is an important signal for governments to intervene with policies and regulations that alter the market place so that the population can gain greater long-term utilities [17,21]. Governments commonly enact policies which curtail commercial activities and individual choices in order to improve health outcomes such as reductions in the road toll, smoking, illicit drug use, workplace injuries and so on. The case for government policy intervention in the commercial market place to achieve health, health equity and quality of life gains by reducing obesity is strong, particularly for children.

## Current government responses to obesity

In an ideal world, governments would have been monitoring population obesity trends and have acted early to implement the actions needed to halt and reverse the obesity epidemic. However, this is not the common reality and, indeed, only a handful of countries have monitoring systems in place to detect changes in the prevalence of obesity and its risk factors. For example, the most recent national surveys of children in Australia were 1985 and 1995 [22], and New Zealand had no national childhood data until 2003 [23]. For an epidemic that started a quarter of a century ago and is now probably the single biggest threat to the health of Australian and New Zealand children, this is indeed a tardy public health response.

It was only when the childhood obesity epidemic started appearing regularly in the media in about 2002–2004 [24] that governments in Australia and New Zealand started to consider action and, to date, their progress has been slow. National plans for action were developed and published [25,26] over 2004–2006 but have yet to be resourced for implementation and the development of the policy backbone has been piecemeal and slow. The action taken to date in Australia has been dominated by television advertisement campaigns run by state and federal governments (but which fall far short of effective social marketing) and community-level programs. The whole-of-community demonstration projects in Australia and New Zealand have the potential to make progress at the community level and these could be substantially enhanced by stronger state and national policies. The best example of policy leadership has come from the NSW, Queensland, and, more recently, New Zealand government policies on food sold in schools [27-29]. A nutrient profiling system has underpinned these policies to identify the 'red light' foods that can only be sold once or twice a term in the schools.

## The roles for governments

We are now at a point where governments are belatedly aware of the threat that rising obesity poses to population health as well as to society's economic well-being and the natural environment [30]. The awareness of the size and complexity of the problem is also evolving into an awareness of the need for multiple actions to achieve a high enough 'dose of solutions'. There is widespread agreement that a multi-sectoral response will be needed from governments, the private sector, civil society and the public [31]. Within this societal approach, what are the roles of governments? Table 1 outlines the four broad roles for governments in the efforts to turn the obesity epidemic around: leadership, advocacy, funding, and policy. The table also provides the rationale to demonstrate how important the roles of government are and some examples illustrate some of the concrete actions that can be taken. Government policy is fundamental as an early driver for change across society and this is the main focus of the remainder of this paper.

**Table 1:** Roles of government in obesity prevention

**Action area**	**Description**	**Rationale**	**Examples**
**Leadership**	∘ Providing a visible lead ∘ Reinforcing the seriousness of the problem ∘ Demonstrating a readiness to take serious action	∘ All societal change needs strong leadership ∘ The role of governments is central, powerful and carries sufficient authority to stimulate a sustained multi-sector response ∘ Government voices speak loudly about problems ∘ Government actions speak louder about solutions	∘ Being visible in the media ∘ Role modelling healthy behaviours (at an individual level) ∘ Role modelling healthy environments (at a government agency level) ∘ Creating mechanisms for a whole-of-government response to obesity ∘ Lifting the priority for health (versus commercial) outcomes
			
**Advocacy**	∘ Advocating for a multi-sector response across all societal sectors (governments, the private sector, civil society, and the public)	∘ Solutions will need to involve many sectors within governments and all sectors outside government ∘ Authoritative mechanisms will be needed to achieve cross-sectoral collaboration and coordination	∘ Advocating to the private sector for corporate responsibility around marketing to children ∘ Creating a high-level taskforce to oversee and monitor multi-sector actions ∘ Encouraging healthy lifestyles for individual and families
			
**Funding**	∘ Securing increased and continuing funding to create healthy environments and encourage healthy eating and physical activity	∘ Changing environments requires funding ∘ Social marketing and programs require funding ∘ Supporting actions (eg training, research, evaluation, monitoring) require funding ∘ Public good funding comes mainly from government sources	∘ Establishing a health promotion foundation (eg using an hypothecated tobacco tax) to fund programs and research ∘ Moving from project funding to program and service funding for obesity prevention ∘ Creating centres of excellence for research, evaluation and monitoring
			
**Policy**	∘ Developing, implementing, and monitoring a set of policies, regulations, taxes, and subsidies that make environments less obesogenic and more health promoting	∘ Most behaviours are heavily influenced by environmental factors (physical, economic, policy, socio-cultural) ∘ Changing environments often requires policy drivers ∘ Education-based approaches are weak without supportive environments	∘ Banning the marketing of unhealthy foods to children ∘ Subsidising public transport and active transport more than car transport ∘ Requiring 'traffic light' front of pack labelling of food nutrient profiles ∘ Restricting the sale of unhealthy foods in schools

## The 'policy backbone' to reduce obesogenic environments

As identified by WHO in the Global Strategy on Diet, Physical Activity and Health [31], the impetus for change needs a critical level of political leadership and some defined policy directions to address obesity. The policy instruments include the 'soft paternalism' approaches of social marketing, health promotion programs and government advocacy for changes in individual and organisational behaviour as well as the 'hard paternalism' options of laws, regulations, enforceable policies, and fiscal instruments [32]. The softer instruments are preferred by most governments, but there are growing calls for the law to used to help tackle obesity [33]. It is entirely possible that 'softer' interventions, such as health education, may increase health inequalities if it they are picked up more by higher-income people than lower-income people. Laws and regulations, on the other hand, tend to be applied across the board, so state policies banning vending machines in schools should at least not increase inequalities and in fact may reduce inequalities if the schools in poorer areas had more vending machines in the first place.

If the harder end of policy is to be applied, what would a substantive, effective 'policy backbone' for reducing obesity look like? Clearly, a comprehensive backbone would cover the policy action at all levels of government. The private sector and non-government sector could also contribute policy initiatives, but in reality, most of the policy will need to come from governments. Examples of analysis grids for policies which may influence obesity are set out in an accompanying paper [34] and it is clear that there are many policy barriers to healthy eating and physical activity and many gaps that health-promoting policies could fill. Importantly, virtually all of the hard policy options are directed at the environment (making the healthy choice the easy choice) and virtually all of the policies that directly target the population are softer options (encouraging people to make the healthy choice). This puts lie to the perception, emphasized by some private sector interests, that government policies will result in a 'nanny state' – implying that the state will be telling people what they can and cannot eat. Governments have not shied away from requiring certain behaviours of their citizens when the public health threat is high – seatbelts, workplace safety, smokefree areas, and illicit drugs are common, everyday examples. But requiring certain eating and physical activity behaviours to prevent obesity or chronic diseases is highly unlikely to happen.

Some of the policy options will be making existing laws and regulations less obesogenic. For example, an unintended consequence of regulations prohibiting the importation of fruit such as bananas and apples into Australia may mean that consumers pay more (and thus presumably eat less) of these foods. Conversely, subsidies on sugar and plant oil production will make energy dense foods cheaper (and thus stimulate consumption). People's consumption patterns are very price dependent [35,36]. An 'obesity impact assessment' may be a form of health impact assessment that needs to be applied to such policies at the time of their formulation.

Many government policy options have significant commercial implications and therefore it is not surprising that some of these proposals (such as banning junk food marketing to children [37]) encounter heavy opposition from the corporate sector. This opposition, which is currently being led by the food and advertising sector but will no doubt be joined by the automobile and oil companies in the future, is one of the major hurdles that governments face in making regulations for obesity prevention. 'Reducing red tape' has been a strong policy direction from Federal and State governments for some years, so making more regulations will also run counter to this. For some policy interventions, such as the universal measurement of body mass index (BMI) in children and sending a 'BMI Report Card' back to parents [38], there may be public opposition to contend with as well.

## Policy lessons from other epidemics

Tackling many other public health epidemics and threats in the past has required a backbone of hard policies around which the softer options can work to amplify their effectiveness [39,40]. Tobacco control is the classic case where taxation, advertising bans, and smokefree environments legislation served as the drivers for change with quit programs, social marketing and education providing added value [41,42]. Reducing the road toll and injuries has required a substantial number of laws and regulations around speed, seat belts, vehicle safety, drink driving and so on to which has been added social marketing and education campaigns and a large amount of vehicle safety enhancements [43]. Infectious disease control is a highly regulated public health endeavour, as is the control of poisons and toxins. Reductions in cardiovascular diseases have been dominated by medical interventions [44] which has proved to be an effective, albeit very expensive approach. Legal and policy interventions are available to reduce cardiovascular diseases [45] but they tend to remain in the realm of 'could do' rather than 'have done' options.

Many parallels have been drawn between other epidemics and the obesity epidemic. Tobacco control is the usual analogy [46] and this is rebutted by the food industry with the statement that food and tobacco are completely different. It is true that the products are completely different but the observed patterns of corporate responses to the public health pressure for regulations and the required spectrum of solutions for the epidemics, including regulatory and fiscal interventions, are remarkably similar.

Even though legislation for obesity prevention could not be directly aimed at eating and physical activity behaviours, any 'rule-based' approach (even at the level of school or home rules) is likely to be a powerful way of changing social norms and attitudes. For example, a policy banning high fat or sugar food and beverages from school canteens, such as the ones being enacted in some states in Australia and in New Zealand, can be expected to accelerate the transition in norms from canteens full of foods high in fat, sugar and salt to canteens with foods that match those promoted in school's nutrition curriculum. While only a few percent of children's total yearly energy intake comes from the school canteen, having visible icons of healthy food are likely to be very important in influencing eating patterns outside school [47]. Such policy interventions could be considered 'lighthouse' interventions because they cast their light far and wide and show children and parents the way forward for healthy eating.

## Combining obesity with other policy imperatives

Obesity is currently attracting public and political attention but this may not be a lasting phenomenon. Indeed, the stigma associated with being obese means that the public constituency agitating for change is quite small. There is not a groundswell of overweight and obese people calling for action – the pressure is predominantly coming from the professional sector. Therefore, it will be important for obesity prevention advocacy to combine with other like-minded 'movements' to get policy action. Interventions which promote healthy eating or physical activity but are enacted for other reasons could be considered 'stealth interventions' [48]. Three such 'movements' centre around climate change [49], congestion in cities [50], and the 'New Nutrition Science' [51] which seeks to incorporate environmental outcomes, such as sustainability and minimising degradation, into the debate and science around nutrition and the food supply.

Policies to reduce greenhouse emissions, such as corporate and individual carbon trading, would be powerful stealth interventions for obesity prevention [49]. Congestion taxes [50], car-free cities [52], public transport growth [53] and other urban planning options [7] will have increased physical activity as a beneficial side effect and thus contribute to obesity prevention. Reducing the carbon cost of food could also have an effect on energy intake since many of the energy dense foods which promote obesity tend to be more processed, packaged foods – in other words, higher in carbon costs.

## Conclusion

Government policy leadership will be needed to accelerate effective action to reduce obesity and its associated inequalities. The suite of interventions will have to include some 'hard paternalism' policy options such as legislation and regulation to make human environments less obesogenic. The calls for action from public health and community advocates in many countries are strong, especially around childhood obesity. There are rare examples of real political leadership being added to the mix and, in those circumstances, real progress can be made [38]. In most places, however, the foremost challenge is to achieve that political leadership. All of the usual processes of political advocacy will be needed in this endeavour but there is also substantial overlap between the solutions for obesity and the solutions for environmental sustainability, reduced congestion, and urban liveability. Collaborations across these movements will create greater pressure for change and greater coordination of action. Indeed for obesity, it may be that the 'stealth interventions' for environmental sustainability prove to be powerful forces for reducing obesity.

## Competing interests

The author declares that he has no competing interests.
